# Changes in expression of oestrogen regulated and proliferation genes with neoadjuvant treatment highlight heterogeneity of clinical resistance to the aromatase inhibitor, letrozole

**DOI:** 10.1186/bcr2611

**Published:** 2010-07-20

**Authors:** William R Miller, Alexey Larionov

**Affiliations:** 1 Breast Research Group, University of Edinburgh, Western General Hospital, Crewe Road South, Edinburgh EH4 2XU, UK; 2 Current address: 2 Stoneycroft Road, South Queensferry, EH30 9HX, West Lothian, UK; 3 Edinburgh Breakthrough Breast Research Unit, Edinburgh University, Western General Hospital, Crewe Road South, Edinburgh EH4 2XU, UK

## Abstract

**Introduction:**

Clinical resistance is a major factor limiting benefits to endocrine therapy. Causes of resistance may be diverse and the mechanism of resistance in individual breast cancers is usually unknown. The present study illustrates how changes in the expression of proliferation and oestrogen-regulated genes occurring during neoadjuvant treatment with the aromatase inhibitor, letrozole, may define distinctive tumour subgroups and suggest different mechanisms of resistance in clinically endocrine resistant breast cancers.

**Methods:**

Postmenopausal women with large primary oestrogen-receptor (ER)-rich breast cancers were treated neoadjuvantly with letrozole (2.5 mg daily) for three months. Clinical response was determined by ultrasound changes in tumour volume. Tumour ribonucleic acid (RNA) from biopsies taken before, after 14 days and after three months of treatment was hybridized on Affymetrix U133A chips. Changes in expression of KIAA0101, TFF3, SERPINA3, IRS-1 and TFF1 were taken as markers of oestrogen regulation and those in CDC2, CKS-2, Cyclin B1, Thymidine Synthetase and PCNA as markers of proliferation.

**Results:**

Fifteen tumours with < 50% volume reduction over three months of treatment were classified as being clinically non-responsive. Gene expression changes after 14 days of treatment with letrozole revealed different patterns of change in oestrogen regulated and proliferation genes in individual resistant tumours. Tumours could be separated into three different subgroups as follows: i) nine cases in which both proliferation and oestrogen signalling signatures were generally reduced on treatment (ii) four cases in which both signatures were generally unaffected or increased with treatment and (iii) two cases in which expression of the majority of oestrogen-regulated genes decreased whereas proliferation genes remained unchanged or increased. In 14 out of 15 tumours, RNA profiles were also available after three months of treatment. Patterns of change observed after 14 days were maintained or accentuated at three months in nine tumours but changes in patterns were apparent in the remaining five cancers.

**Conclusions:**

Different dynamic patterns of expression of oestrogen-regulated and proliferation genes were observed in tumours clinically resistant to neoadjuvant letrozole, thus illustrating heterogeneity of resistance and discriminating molecular sub-classes of resistant tumours. Molecular phenotyping might help to direct circumventing therapy suggesting the targeting of specific pathways in different tumour subtypes.

## Introduction

Endocrine therapy is a major treatment modality for breast cancer but its utility is limited by both primary and acquired resistance [1-3]. Current selection for the treatment is based on presence of oestrogen receptors (ER) in the tumour [4-7]. The growth of many ER-positive tumours slows in response to oestrogen deprivation; however, some tumours fail to respond, despite the possession of oestrogen receptors [8,9]. Moreover, the majority of patients, who initially respond to treatment, develop resistance later. At present, there are no rational targeted therapies to overcome endocrine resistance and no clinical markers to predict resistance in ER-positive breast tumours. Patient management would benefit from accurate identification of (i) tumours most likely to respond to treatment and (ii) the mechanisms of resistance in individual non-responsive cancers. In order to address these issues, a neoadjuvant therapy with letrozole, a specific aromatase inhibitor which reduces endogenously synthesised oestrogen [10], has been employed [11]. The study reports molecular profiles in sequential biopsies taken from breast tumours during the course of treatment. This design allows assessment of dynamic changes in gene expressions in individual tumours. Present analysis concentrates on the heterogeneity of gene expression changes within the resistant tumours focussing on genes associated with proliferation and oestrogen signalling.

## Materials and methods

### Patients

All patients were postmenopausal women presenting to the Edinburgh Breast Unit with large primary ER-rich (Allred score > 5) breast cancers but without evidence of distant metastatic disease. Informed consent was obtained for inclusion in the study which had been approved by the local ethics committee (LREC 2001/8/80 and LREC 2001/8/81). Neoadjuvant treatment was with letrozole (Femara, [Novartis Pharma AG, Basel, Switzerland] 2.5 mg daily) for three months [11]. Clinical response was based on changes in tumour volumes over three months determined from ultrasound measurements (performed by a single operator). Tumours with < 50% reduction in volume were classified as non-responders. Demographics of patients with clinically resistant tumours are summarized in Table 1.

**Table 1:** Patient and tumour characteristics

Age at diagnosis	Years
Median	79
Range	63 to 86

**Tumour size**	**n (%)**

T2	11 (73)
T3	1 (7)
T4	3 (20)

**Lymph node status**	**n (%)**

+ve	2 (13)
-ve	13 (87)

**Histological grade**	**n (%)**

1	1 (7)
2	9 (60)
3	4 (26)
Unknown	1 (7)

**Progesterone receptor**	**n (%)**

+ve	11 (73)
-ve	4 (27)

**HER2**	**n (%)**

+ve	2 (13)
-ve	13 (87)

### Tumour processing and RNA extraction

Multiple core biopsies were taken with a 14 gauge needle before and after 10 to 14 days of treatment. In most of the cases the tumour was also available after three months of treatment. Tissue samples were immediately snap-frozen and stored in liquid nitrogen. Frozen sections were taken to confirm the presence of cancerous tissue. Biopsies in which the malignant component comprised at least 20% of the section area were pulverised using U2 micro-dismembranator U (Braun Biotech, Melsungen, Germany). Total RNA was extracted from the frozen tissue powder using TRI-reagent (Sigma, Poole, Dorset, UK). Before microarray analysis, the extracted RNA was further purified on RNeasy mini columns (Qiagen, Crawley, West Sussex, UK).

### Microarray analysis

RNA (500 ng) was subject to two rounds of amplification [12]. The resulting cRNA was converted to double-stranded DNA and biotinylated cRNA was generated using the Enzo kit (Affymetrix, Santa Clara, California, USA). Biotinylated cRNA was fragmented and hybridized on Affymetrix (Santa Clara, CA, USA) HG_U133A chips as described in the standard protocol outlined in the Gene Chip Expression Analysis Technical Manual (Affymetrix). Microarrays were scanned with an Affymetrix 3000 laser scanner. Raw expression values from Affymetrix' CEL files were normalised using Robust Multichip Average methodology [13-16]. The method adjusts for background noise on chips and summarizes data into expression values, one number per gene per sample. Primary microarray data are available from the Gene Expression Omnibus [17] with series numbers [GEO:GSE5462] and [GEO:GSE20181]).

### Genes associated with oestrogen regulation and proliferation

Marker genes classically associated with oestrogen regulation were *KIAA0101 (202503_s_at), TFF3 (204623_at), SERPINA3 (202376_at), IRS1 (204686_at), TFF1 (205009_at) *and those associated with cell proliferation were *CDC2 (203213_at), Cyclin B1 (214710_s_at), CKS2 (204170_s_at), TYMS (202589_at), PCNA (201202_at*). They were chosen because of previous literature indicating these associations [18-26] and having been demonstrated to be detectably expressed by this series of breast cancers [27]. They included oestrogen regulated genes resulting from interactions at both ERE and AP-1 sites. Single probes were present on the chip for all genes apart from *KIAA0101*, *CDC2 *and *TYMS *for which the most specific probe was selected. All expression calls were positive before treatment and > 90% overall.

### Real-time quantitative PCR

Microarray measurements for four of the studied genes were verified by real time PCR. These included two oestrogen regulated (*SERPINA3 *and *TFF1*) and two cell cycle associated (*CCNB1 *and *CDC2*) genes. In brief, mRNA was converted to cDNA using oligo-dT primers and SS-III reverse transcriptase (Invitrogen), according to the manufacturer's instructions. Real time PCR was run using Quantitect SYBR-green PCR mix (Qiagen) on Opticon Monitor 2 machine (Biorad MJR, Bio-Rad Laboratories Ltd, Hemel Hempstead, Hertfordshire, UK) as described [28]. Expression was normalised by geometric mean of three stably expressed reference genes [29,30]. Primer sequences designed using Primer3 software (Enzo Life Sciences, Exeter, UK) [31] are shown in Table S1 in Additional file 1.

## Results

### Clinical response/resistance

A total of 58 tumours were analysed for genomic changes [27]. Of these, clinical response was not assessable in six because of inconsistencies in assessments by calipers, ultrasound, mammography and microscopy. The remaining 52 tumours were classified as 37 (71%) responders and 15 (29%) non-responders [11]. The latter tumours represent the cases analysed in the present paper. Histologically, all were of no special type. Other demographics are listed in Table 1.

### Oestrogen receptor scores

Eleven tumours had an Allred score of 8: the remaining scored 7 before therapy. Treatment produced no or only minor effects (+/- 1) on staining apart from a single tumour in which scores fell successively from 8 to 7 to 5 with increased time of therapy. These results are similar to those published previously for the extended group including responsive tumours [32].

### Changes in gene expression associated with therapy in clinical non-responders

**Figure 1:**
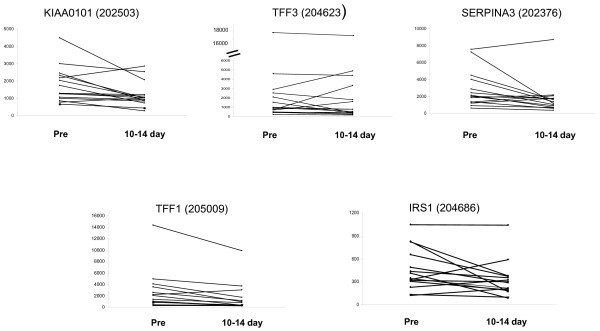
**Levels of oestrogen-regulated genes before (pre) and after treatment with Letrozole (10 to 14 days)**.

**Figure 2:**
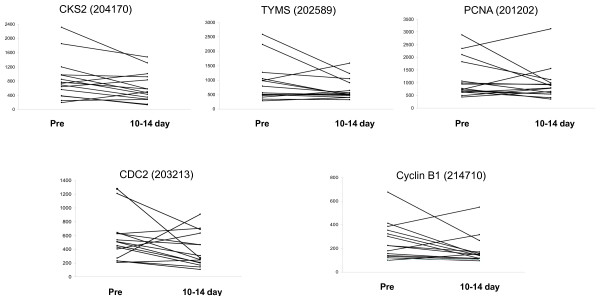
**Levels of proliferation-associated genes before (pre) and after treatment with Letrozole (10 to 14 days)**.

**Figure 3:**
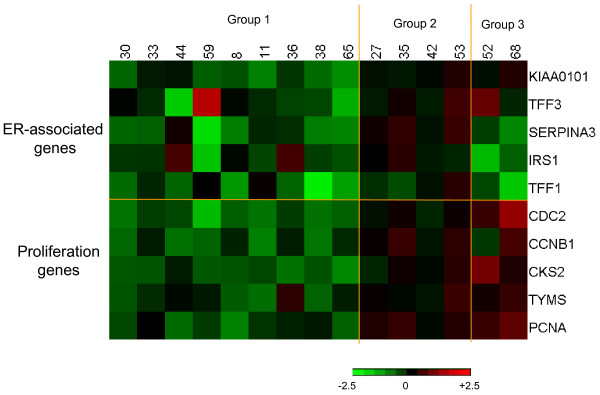
**Heterogeneity of endocrine-resistant tumours**. Heatmap illustrates changes after 10 to 14 days of treatment with Letrozole: Green represents a decrease in mRNA expression, red represents an increase in mRNA expression, brightness of colour corresponds to the degree of changes. Scale in log to the base 2.

Changes in studied markers after 10 to 14 days of treatment are illustrated in Figures 1 and 2. Although the general trend for oestrogen regulated genes was a decrease in expression (Figure 1), group differences with treatment were not statistically significant apart from *SERPINA3 *(*P *= 0.045 by paired Wilcoxon rank test). Neither were there consistent changes (Figure 2) nor significant differences with treatment in individual genes associated with proliferation. Patterns of gene changes occurring after 10 to 14 days of treatment are illustrated in a heat map (Figure 3). This highlighted differences between individual resistant tumours, which could be sub-grouped according to whether (i) both markers of oestrogen regulation and proliferation were decreased (nine cases - Group 1), (ii) all markers were only marginally changed (four cases - Group 2) and (iii) markers for oestrogen regulation were decreased whereas those for proliferation were unchanged/increased (two cases - Group 3).

### Changes in molecular phenotype at three months

**Figure 4:**
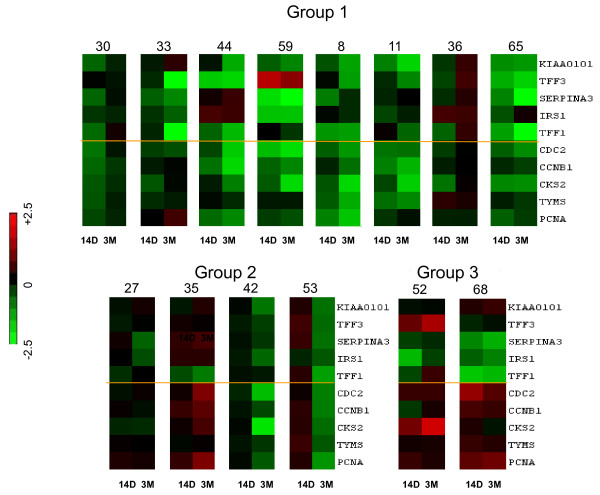
**Sequential changes in genes expression in individual tumours**. The left bar represents changes between pre-treatment and 14 days (14 D) and the right bar represents changes between pre-treatment and three months (3 M).

Tumours were also available after three months of treatment. Microarray analysis was successfully performed on 14 of these specimens. Patterns of changes between (i) Day 0 and 10 to 14 days and (ii) Day 0 and three months of treatment are illustrated in a heat map in Figure 4. Of the nine cases in which 10-to-14-day treatment was associated with reduced expression of both oestrogen regulated and proliferation markers (Group 1), microarray results at three months were available for eight cases. In five of them expression of the markers continued to be reduced in comparison with pre-treatment levels and often were further suppressed in relation to the 10-to-14-days; in two cases (#30 and #36) both oestrogen-regulated and proliferation marker mRNAs returned toward pre-treatment values; and in the remaining tumour (#33) oestrogen regulated markers were further suppressed at three months whereas proliferation markers returned toward pre-treatment levels. Of the four cases from Group 2 (marginal changes in the studied genes at 14 days), two tumours still had only marginal changes at three months, whereas two cases (#42 and #53) displayed clear reduction in expression of the genes. Finally both tumours with differential changes in expression of oestrogen regulated and proliferation genes (group 3) maintained this phenotype at three months as well as at 14 days.

### Quantitative PCR measurements

The expression of *SERPINA*, *TFF1*, *CCNB1 *and *CDC2 *were also measured by qRT-PCR in 42 of the tumour samples assayed by microarray. Results for the correlation coefficients and corresponding significance values are summarized in Table S2 in Additional file 2. Highly significant positive correlations were detected between the two methodologies for each gene (including responding cases from the total database greatly increases the number of sample comparisons and the *P-*values are < 10^-15 ^for each gene).

## Discussion

Oestrogen deprivation is a major therapeutic option used to treat hormone sensitive breast cancer [33]. Blockade of oestrogen synthesis using aromatase inhibitors occupies a central role in the management of postmenopausal women with oestrogen receptor-positive tumours. Agents such as letrozole, anastrozole and exemestane have great potency and specificity [10,34-36] and also can be used to examine the molecular effects of oestrogen deprivation in breast cancers *in vivo *[27,37,38]. However, not all ER-positive tumours respond to aromatase inhibitors [1,33] and there is, therefore, a need for molecular markers which predict response to treatment and elucidate molecular mechanisms associated with different mechanisms of resistance. An important element of the latter is to determine the effects of treatment on expression of oestrogen regulated genes and proliferative pathways in tumours resistant to treatment. Given the potential diversity of resistance mechanisms, there are major advantages in using a neoadjuvant protocol in which effects of treatment may be monitored and correlated with clinical response in individual tumours. An additional strength of neoadjuvant therapy is that the accessibility of the primary breast cancer means that clinical response may be accurately assessed by sequential measurement of tumour volume and the cancer may be serially biopsied to monitor changes in gene expression. These characteristics mean that meaningful analyses can be performed by studying relatively small numbers of tumours.

The present paper is based on expression changes in a gene subset derived from a genome-wide microarray analysis of biopsies of 52 breast cancers including the focus subgroup of 15 tumours, clinically resistant to neoadjuvant treatment with letrozole. Expression of *SERPINA3, TFF1, CCNB1 *and *CDC2 *was also analysed by quantitative RT-PCR. For all these validated genes, PCR and microarray measurements showed highly significant positive correlations on both the total dataset and the subset of the resistant cases. However, despite the highly significant correlations there was a small number of gross outliers. (This may account for the paradoxical changes seen in the *TFF3 *found in tumour #59, which were increased in expression at both 14 days and three months.) To account for occasional outlying results, we used five genes for each of studied pathways. Thus, expression of *KIAA0101*, *insulin receptor substrate 1*, *SERPINA3 *and *trefoil factors 1 *and *3 *is either induced by oestrogen or reduced by oestrogen deprivation/antioestrogens in experimental systems [19-22,39-41] and has been used to denote functional oestrogen signalling. It is worth commenting that although the expression of these genes is down-regulated with letrozole treatment, similar differential changes were observed in genes such as *COLEC12 *and *HTRA1 *which were up-regulated by letrozole treatment (data not shown). *CDC 2, Cyclin B1, CKS2, TYMS *and *PCNA *are intimately involved in proliferative processes and are often down-regulated by oestrogen deprivation in oestrogen-responsive systems *in vitro *[19,26,42]. As a result of measuring these markers in sequential biopsies during treatment, it was possible to show variation in molecular responses between individual endocrine-resistant cases. Thus, by using differences in patterns observed after short-time treatment (10 to 14 days) tumours could be allocated to subgroups, namely: (i) cases in which both markers of oestrogen regulation and proliferation were generally decreased, (ii) cancers in which expression of most of the examined genes was only marginally affected and (iii) those in which changes in oestrogen-regulated and proliferation genes were disconnected: markers for oestrogen regulation were decreased whereas those for proliferation were unaffected/increased by treatment. This would be in keeping with the diversity of mechanisms by which it has been proposed that tumours may become resistant to aromatase inhibitors [8,43] and other endocrine therapies [19,44-46]. In the majority of cases analysed in this study, clinical resistance was paradoxically associated with reduction in expression of most oestrogen-regulated and proliferation genes (Figure 3: Group 1). Additionally, all seven tumours in this group which were PgR-positive displayed a reduction in staining intensity and score with treatment. All had a reduction in Ki67 score (apart from a case with low proliferation at the outset). Median Ki67 scores were 15.6 before treatment, 6.0 after 14 days and 5.6 after three months of treatment. Thus, whilst these tumours are categorized as clinical non-responders, they do react to oestrogen deprivation at molecular and proliferative levels.

The major issue to clarify is why molecular and proliferative responses associated with oestrogen deprivation by letrozole do not translate into clinical responses. There are several potential reasons. First, it may reflect limitations and inaccuracy of clinical measurements. Current clinical criteria for response assessment are often based on arbitrary empirical thresholds and it may be that clinical measurements do not reflect biological responses. Thus, in the present study tumours have been categorized as *clinically resistant *on the basis of less than 50% reduction in tumour volume. However, nine of these *clinically resistant *tumours still had > 25% reduction. These tumours might have become clinical responders with extended treatment (we have shown that volume reduction continues beyond three months with letrozole [47,48] and other forms of endocrine therapy [49]). In such cases the molecular profiling may complement clinical measurements in response assessment. Another reason for the disparity between molecular and clinical responses is that the molecular phenotypes were transient, and compensatory changes occurred in gene expression. However, evidence for these were observed in only the minority of tumours, and in the remainder the decrease in gene expression was even greater at three months than at 10 to 14 days. Finally, it should be emphasized that treatment did not decrease gene expression to zero and, after therapy, expression is still measurable. Hence, it could be argued that the relative reductions in proliferation are not sufficient to produce a clinical response in the absence of other changes such as an increase in cell death.

Cases in which gene expression was only marginally affected appear to have the classical phenotype of oestrogen insensitivity (Figure 3: Group 2). However, before labelling such tumours as *oestrogen resistant*, it needs to be confirmed that the patients were drug compliant and that 10 to 14 days of treatment reduced both circulating and intratumoural levels of oestrogen. Interestingly, in two of these tumours, gene expression was reduced at three months possibly indicating that endocrine and clinical response might require a more prolonged treatment. Parallel changes were seen in Ki67 staining with a median score of 6.2 before treatment, 5.4 after 14 days and 3.4 after three months' treatment.

A differential phenotype in which expression of oestrogen-regulated genes was mostly reduced but that for proliferation genes was generally increased was observed in two tumours (Group 3). Interestingly, this was also evident in the protein staining for progesterone receptor (which was positive and decreased with treatment in both tumours) and Ki67 (mean value before treatment 12.3 and 16.6 after 14 days treatment). The disconnection between expression of oestrogen signalling and proliferation genes was not transient and was observed at both 10 to 14 days and three months (although Ki67 staining was markedly decreased at three months in both cases - mean score 3.6). The most obvious explanation for this phenomenon is that whilst oestrogen regulated genes are still controlled by oestrogen, proliferation (and growth) is determined by other non-oestrogenic pathways. Of note, these two tumours did not carry HER2 amplification.

The above discussion reflects the complexity and heterogeneity of molecular changes occurring within a relatively small series of clinically resistant tumours following neoadjuvant treatment with the aromatase inhibitor, letrozole. This would be compatible with the diversity of molecular mechanisms leading to resistance. It should be noted that this heterogeneity is independent of ER score which was not, or marginally, changed with treatment. Furthermore, since changes in genes classically associated with oestrogen regulation are frequently seen with treatment, primary clinical resistance to letrozole should not be equated to hormone-insensitivity at molecular level. Indeed it could be that in some cases specific non-canonical molecular changes produced by treatment may be the cause of clinical resistance. The challenge remains to explain why marked reductions in proliferation do not always translate into clinical response (and, as a consequence, do not provide robust markers for response prediction in individual patients). A greater understanding of the molecular processes involved and a systematic study of factors such as, i) whether circulating and intratumoural oestrogen are reduced, ii) the patency of oestrogen signalling pathways, iii) the degree to which proliferation is suppressed, and iv) the involvement of other pathways signalling for growth and cell survival in individual tumours appear to be important and necessary steps by which to optimise treatment with aromatase inhibitors such as letrozole.

## Conclusions

These data demonstrate that dynamic assessment of oestrogen signalling and proliferation during treatment with letrozole can identify distinctive molecular subgroups within breast cancers clinically resistant to endocrine therapy.

## Abbreviations

ER: oestrogen-receptor.

## Competing interests

The authors declare that they have no competing interests.

## Authors' contributions

WRM designed the study protocol and was the principal investigator. WRM and AL jointly collected, analysed and interpreted the data, and wrote and edited the manuscript.

## Supplementary Material

Additional file 1 **Supplementary table S1**. PCR primers' sequences. Click here for file

Additional file 2 **Supplementary table S2**. Correlations between PCR and Microarray measurements. Click here for file
