# Supplementary material for: Changes in expression of oestrogen regulated and proliferation genes with neoadjuvant treatment highlight heterogeneity of clinical resistance to the aromatase inhibitor, letrozole
Source: Breast Cancer Res. 2010 Jul 20;12(4):R52. doi: 10.1186/bcr2611 (PMC2949641; doi:10.1186/bcr2611)
Supplement: Additional file 1 — Supplementary table S1. PCR primers' sequences. [file bcr2611-S1.DOC]

# Table S1 - PCR primers’ sequences

| Gene | Forward | Reverse | Comment |
| --- | --- | --- | --- |
| CCNB1 | CATGGTGCACTTTCCTCCTT | CAGGTGCTGCATAACTGGAA | Target |
| CDC2 | TGGCCAGAAGTGGAATCTTT | AAATTCGTTTGGCTGGATCA | Target |
| TFF1 | TTGTGGTTTTCCTGGTGTCA | AAAATTCACACTCCTCTTCTGGA | Target |
| SERPINA3 | CTCCCAGAGACCCTGAAGC | GCCCAGCTGGAGAAGTATGT | Target |
| KIAA0674 | GGGCAACTCTGAAAGCTCTG | CTTCATCCCAGCCAGTCAAT | Reference |
| PUM1 | CGTGGTCCAGAAGATGATTG | TACGAAGAGTTGCGATGTGG | Reference |
| TBP | GGGGAGCTGTGATGTGAAGT | CCAGGAAATAACTCTGGCTCA | Reference |
