# Supplementary material for: Changes in expression of oestrogen regulated and proliferation genes with neoadjuvant treatment highlight heterogeneity of clinical resistance to the aromatase inhibitor, letrozole
Source: Breast Cancer Res. 2010 Jul 20;12(4):R52. doi: 10.1186/bcr2611 (PMC2949641; doi:10.1186/bcr2611)
Supplement: Additional file 2 — Supplementary table S2. Correlations between PCR and Microarray measurements. [file bcr2611-S2.DOC]

# Table S2 - Correlations between PCR and Microarray measurements

| Gene | Whole Letma2 dataset | | | Non Responders | | |
| --- | --- | --- | --- | --- | --- | --- |
|  | N | R | p | N | R | p |
| SERPINA3 | 172 | 0.81 | <2.2×10^-16^ | 42 | 0.77 | 2.7×10^-9^ |
| TFF1 | 168 | 0.80 | <2.2×10^-16^ | 42 | 0.84 | 5.2×10^-12^ |
| CCNB1 | 172 | 0.66 | <2.2×10^-16^ | 42 | 0.58 | 5.7×10^-5^ |
| CDC2 | 172 | 0.77 | <2.2×10^-16^ | 42 | 0.77 | 2.0×10^-9^ |
